# Supplementary material for: PEGylated graphene oxide elicits strong immunological responses despite surface passivation
Source: Nat Commun. 2017 Feb 24;8:14537. doi: 10.1038/ncomms14537 (PMC5333105; doi:10.1038/ncomms14537)
Supplement: Supplementary Information — Supplementary Figures, Supplementary Tables, Supplementary Methods and Supplementary References. [file ncomms14537-s1.pdf]

Supplementary Methods

Nano-graphene oxide (nGO) configuration setup. To generate an nGO system suitable for simulation, coordinate and CHARMM topology files for a 5 nm × 5 nm bare graphene sheet were first created using VMD's Nanotube Builder plugin[1]. In rough accordance to the Lerf-Klinowski model[2] of graphene oxide, hydroxyl, carboxyl, and epoxide groups were added to the faces and edges of the graphene sheet such that approximately 7% of aromatic carbons were oxidized. Carboxyl groups were added in relative excess to the edges of the nanosheet, which should be more susceptible to over-oxidation in physical systems.

PEG conjugation to nGO. Extended polyethylene glycol chain configurations were generated manually, using the RCSB PDB entry for PEG as a reference for monomer and methylated terminus coordinates. Each chain consisted of 15 ethylene glycol monomers, a methylated terminus, and an amide linkage to the GO nanosheet, resulting in an approximate molecular weight of 762 amu/polymer. This particular polymer length was chosen in approximate correspondence to experimental parameters (MW 2000), truncated somewhat to facilitate tractability in simulations. To complete the nGO-PEG systems, six carboxyl groups (one on each edge and face) were replaced with the amide linkages on each polymer, covalently attaching the PEG chains to the GO nanosheet under consideration.

Force field parameterization. Parameters for PEG monomers and termini were extracted directly from the CHARMM ether force field[3]. In a manner consistent with past work[4], parameters for carboxyl, hydroxyl, and epoxide groups and PEG amide linkages were adapted from similar motifs already existing within the CHARMM27 force field[5]. Specifically, hydroxyl group parameters were based on those of the serine side chain, carboxyl group parameters were drawn from the alanine terminus, epoxide parameters were pulled from the ether force field, and amide linkage parameters were derived from alanine-alanine peptide bond parameters. Limited sets of bond, angle, and dihedral parameters specifically related to graphene/functional group bonds (present in no existing force field, to our knowledge) were defined according to basic chemical principles; such parameters are largely overridden by constraints applied in

our simulations, as discussed below. Charges on each oxidized graphene carbon were adjusted to achieve neutrality where needed.

Free nGO-PEG simulations. To study nGO-PEG in the absence of a membrane, the nGO-PEG system described above (featuring extended PEG chains) was solvated in a 20 × 20 × 10 nm box of TIP3P molecules and ionized with 0.15 M sodium and chloride ions, yielding a system containing nearly 350,000 atoms. To both simplify the simulation setup and emphasize the planar properties of nGO, stringent harmonic restraints were placed on both the graphene nanosheet and terminal carbon atoms attached to oxygen-containing functional groups and PEG chains; forces among restrained atoms were not computed. More specifically, a small scaled force constant of 10 kcal mol^{-1} $Å^{-2}$ was applied to all graphene sheet carbon positions (including those of oxidation sites and those of carbons directly bound to PEG chains), yielding a total of 1,008 restrained atoms. Molecular dynamics calculations were completed using the NAMD[6] simulation package, invoking a Langevin integrator held at constant temperature and pressure (310 K; 1 atm). Standard CHARMM27 force field parameters were employed for all interactions not discussed above; PME electrostatics were used in concert with dispersion interactions subjected to a mutual 1.2 nm real space cutoff. Normal SETTLE constraints were applied to enable the use of a 2 fs time step. Simulation trajectories were extended until the PEG adsorption process was deemed to be complete (after approximately 50 ns); an equilibrated snapshot of the nGO-PEG complex was extracted for use in subsequent simulations and featured in Fig. S10.

Membrane configuration setup. Independently, an 8 nm × 8 nm segment of a pre-equilibrated POPC lipid bilayer was generated using the Membrane Builder plugin in VMD[1]. Additional equilibration simulations were conducted for thoroughness. After solvation in TIP3P and the deletion of water molecules in the transmembrane region, lipid tails were melted (with head groups restrained) for 25 ns. The entire system was then equilibrated without restraint for an additional 25 ns. Force field and simulation parameters were identical to those used above, except that pressure control was only implemented in the direction normal to the membrane

surface (as recommended by the CHARMM developers). Periodic boundaries were also defined such that lipids spanned the transverse boundaries in a smooth fashion. An equilibrated membrane configuration was extracted for use in production simulations.

Production simulations. To generate the configurations used in production simulations, the equilibrated nGO-PEG complex (or, simply the initial nGO sheet) was placed in either an edge-on or face-on configuration 1 nm from the membrane surface. After solvation with TIP3P, deletion of water molecules in the transmembrane region, and ionization, short equilibration runs were conducted with the positions of PEG and membrane atoms fixed for 500 ps and free for another 500 ps. Production runs were then conducted under the same restraints used in free nGO-PEG simulations, employing the same force field and simulation parameters described previously. Once more, pressure coupling was only applied along a membrane-normal coordinate. All systems contained approximately 100,000 atoms, and were simulated for several hundreds of nanoseconds (up to a microsecond) until satisfactory convergence was evident.

Simulation analysis. Center of mass calculations between atom groups of interest were carried out using custom Tcl scripts. Specific interaction energies were computed using the NAMD Energy plugin in VMD.

Reagents and Materials. BD™ Cytometric Bead Array (CBA) Mouse Inflammation Kits, Skim milk were obtained from BD Biosciences. Anti-Integrin β_1, β_2, PVDF membrane and Immobilon™ Western chemiluminescent HRP Substrates were obtained from Millipore. Anti-Integrin α_v, α_L, β_8 was ordered from Biolegend. Anti-Integrin α_1 were provided by Abcam. Halt protease and the phosphatase inhibitor cocktail came from Thermo scientific. RIPA buffer and vinculin were ordered from Sigma. Bovine Serum Albumin was (BSA) ordered from Roche. Pre-stained marker was purchased from Biolabs. GADPH came from the Goodhere Corporation. FAK was ordered from Santa Cruz Biotechnology. Phosphorylated FAK (p-FAK) and horseradish peroxidase-conjugated anti-rabbit IgG were purchased from Cell signaling Technology. Lactate dehydrogenase (LDH) Cytotoxicity Assay Kits,

SDS-PAGE gels, DiO and, NF-κB p65 were ordered from the Beyotime Institute. Alexa Fluor® 488, Alexa Fluor® 647, Opti-MEM Reduced Serum Medium, Ambion® Silencer Select siRNA, and RNAimax reagents were all supplied by Invitrogen. GO and CNT powders were provided by Ding Ma's Group of Peking University. All other reagents were of analytical grade.

Animals and cells. C57BL/6 mice were ordered from Charles River Laboratories (USA). All animal experiments were performed in compliance with the institutional ethics committee regulations and guidelines on animal welfare. Peritoneal macrophages (pMØ) were harvested from the stimulated C57BL/6 mice according to a typical protocol[7], and murine macrophage cell line J774A.1 was supplied by ATCC (American Type Culture Collection). Cells were cultured with DMEM cell culture medium added with penicillin (100 U mL^{-1}), streptomycin (100 U mL^{-1}), and 10% fetal bovine serum (FBS) in a humidified incubator held at 37 ^{o}C and 5% CO_2.

Synthesis and characterization of PEGylated carbon materials. To exclude the possibility of complications brought by metal impurities in GO samples, treatments based on those developed in a previous paper[8] were applied here. To remove heavy metals in GO samples, residual $KMnO_4$ and MnO_2 were first cleared with 3% H_2O_2 solution. After washing the product repeatedly with 5% HCl solution and removing all sulfate ions, we finally adjusted the GO solution to neutrality. Through the above washing procedure, all of *in vitro* experiments could be carried out in the presence of relatively benign GO. The metal content of GO before/after washing was measured by WFX-130B Atomic Absorption Spectroscopy (AAS, Beijing Beifen-Ruili) and the corresponding results are presented in Table S1.

nGO-PEG. PEGylation procedures were mainly drawn from our previous report[9]. First, EDC (20 mM) was added into the pristine nGO suspension (~500 μg mL^{-1}) and sonicated for 15 min. Immediately, mPEG-NH_2 (10 mg mL^{-1}) was added and allowed to react overnight. The final product (nGO-PEG) was harvested by centrifugation at 70,000 g after repeated washing by DI water.

Supplementary Note 12: Carbon Spheres. Referring to our previous report[10],

1.08 g glucose was dissolved in 12 mL water to form a clear solution after ultrasonication. The solution was then transferred into a 15 mL high temperature reactor and the brownish black solution was observed when hydrothermally treated at 180 ^{o}C for 8 h. Pure carbon spheres (CS) were obtained through repeated centrifugation in water and alcohol. The concentration of the CS solution was defined by weighing the sample after drying at 70 ^{o}C for 6 h.

The construction method of PEGylated carbon materials was similar to that used for nGO-PEG, as described above. The PEG content of nGO-PEG (Fig. S1, Fig. S2) was analyzed by Escalab 250Xi X-ray photoelectron spectroscopy (XPS, Thermo) and TG-DTA6300 Thermogravimetry (TG, NSK). The morphologies of nGO, CS and CNT were imaged by Dimension FastScan (AFM, Bruker), JSM-6700 Scanning Electron Microscopy (SEM, JEOL) and JEM-1400 Transmission Electron Microscopy (TEM, JEOL), respectively. The zeta potentials of CS/CS-PEG and CNT/CNT-PEG were determined using a Zeta Sizer (Nano Series, Malvern).

The approximate surface areas of CNTs, CSs and nGOs were calculated using the following equations: $S_{CNT}=\pi\times d\times l$ (d=20 nm, l=4 μm), $S_{CS}=4\pi\times r^2$ (r=100 nm), $S_{nGO}=2\times d^2$ (d=200 nm, assumed to be a square). The resultant ratios of surface areas were thus S_{CNT}: S_{CS}: S_{nGO}=3.14: 1.57: 1; we used these ratios to normalize nanomaterial doses among the different carbon materials.

Detection of Cytokine secretion. A CBA Mouse Inflammation Kits were employed to detect cytokine secretion. pMØ cells were stimulated with different GO solution/carbon spheres solutions at the setting time (6h, 12h, 24 h, and 48 h) or dosage (10 μg mL^{-1} and 40 μg mL^{-1}). Cell supernatant collection followed, and cytokine secretion detection proceeded via CyAn ADP flow cytometry (FACS, Beckman Coulter), measuring the levels of IL-6, IL-10, IL-12, TNF-α, MCP-1, and IFN-γ.

Interaction study between nGO complexes and cells. pMØs were seeded (1×10^5 mL^{-1}) in a Petri dish and incubated with nGO complexes at 10 and 40 μg mL^{-1} for 24 h. nGO complex imaging was performed under a flow cytometry PE-Cy7 channel using graphene's intrinsic photoluminescence. The cytoskeleton and nuclei were

subsequently separately stained with rhodamine-phalloidin (green pseudocolor in images) and Hoechst, respectively, for 20 min at room temperature. Images were captured with an Ultraview VoX cell imaging system (PerkinElmer) and the corresponding nuclear parameters were analyzed in a Columbus analysis system (PerkinElmer).

TEM characterization of membrane morphologies treated with nGO-PEG. pMØ cells were seeded on slides in 6-well plates and coincubated with nGO-PEG for 24 h. The culture medium was then washed twice with cold PBS. Samples were fixed in 2.5% glutaraldehyde for 2 h at room temperature, then rinsed with 0.1 M PB thrice and kept at 4 °C. Afterwards, samples were post-fixed, serially dehydrated with ethanol, and embedded in Epon. Finally, serial sections were cut on a Reichert Ultracut microtome (Leica), and electron micrographs were taken using a JEM-1400 Transmission Electron Microscope (TEM, JEOL).

Second stimulation assay. pMØ cells were stimulated with 10 μg mL^{-1} nGO-PEG solutions at the setting time (12 h, 24 h, 48 h). After 12 h, the supernatant was collected (set as ①) and the culture medium was replaced by the fresh water. This nGO-PEG free culture medium was collected again at 24 h (set as ②) and 48 h (set as ③). To investigate the second stimulus level of cells, fresh culture medium was removed again by presenting nGO-PEG contained medium at 24 h, and then the result was analyzed at 48 h (set as ④).

Detection of plasma membrane integrity (LDH Assay). 20 μL LDH release solution was added to the positive well one hour before 24 h of coincubation. Cell culture medium was collected and centrifuged at 3000 rpm for 5 min. An aliquot (120 μL) of supernatant and 60 μL LDH detection reagent were added to a new 96-well plate to quantify the LDH level. After coincubating for 30 min at room temperature without light, absorbances were measured in an Infinite M200 microplate spectrophotometer (Tecan) at 490 nm.

Fluorescence recovery after photobleaching (FRAP). pMØ cells were seeded on

a petri dish overnight to encourage adhesion, and then 2 μL of membrane fluorescent probe DiO (initial concentration 1,000 μM) was added after being dissolved in DMSO. Cells were incubated at 37 ^{o}C and 5% CO_2 for 20 min, and then transferred from the petri dish to the incubator on an Ultraview (Perkin Elmer, America) to monitor the cells under an 100× oil objectiv. Regions of interest in the stained membranes were noted and photobleached using a 488 nm laser operating at 13% power. Single images were then collected at maximum speed. All FRAP data were analyzed with prepackaged analysis software, which can fit the model to experimental data reasonably through a nonlinear curve fit to $f(t) = y + A \cdot e^{-kt}$.

Tracking the trajectories of cells. Adherent pMØ cells were coincubated with or without nGO-PEG on a petri dish overnight. Multiple points of interest were then selected when cells were transferred to the Ultraview incubator. After a period of time, live cell videos were recorded under a bright field and using object-tracking tools. The trajectories of many different individual cells were observed. The trajectory images were quantitatively assessed using a 30 μm reference sphere and aligned with the Ultraview analysis system.

Sample preparation for genechip analysis. pMØ cells were seeded in a 35 mm petri dish at a density of ~ 5000,000 and cultured for 6 h to achieve adhesion. Cells were then induced with 10 μg mL^{-1} nGO-PEG for 12, 24, and 48 h respectively, with each group having three parallel specimens. RNA was extracted from each sample using 0.6 mL trizol reagent per dish, which was sent to Shanghai Gene Corporation for further analysis. Pathway analysis was performed with the help of the KEGG database.

Intergrin antibody blocking experiment. Anti-integrins α_1, α_L, β_1, β_2, and β_8 were added at appointed concentrations and allowed to incubate for 4 h. The supernatant was then replaced with culture medium containing 10 μg mL^{-1} nGO-PEG for 24 h. Finally, the supernatant was removed, and cytokine secretion was measured as described above.

siRNA transfection of integrin β8 gene. Transfection of mouse integrin β_8 siRNA

into pMØs was performed with an RNAimax reagent, according to the manufacturer's instructions. Briefly, using a 6-well plate, 11 μL of integrin β_8 siRNA (No.MSS220603, 20 μM initial concentration and 100 nM final concentration) and 7.5 μL RNAimax were diluted with 100 μL Opti-MEM Medium, respectively. The reagents were coincubated for 5 min before addition to a 2 mL DMEM culture system. The β_8 gene was silenced sufficiently after 48 h of coincubation.

Western blot. For immunoblotting analysis, cell lysates were loaded onto 4-10% SDS-PAGE gels, and subsequently transferred onto a PVDF membrane. The membrane was blocked for 2 h in 5% skim milk/BSA (for p-FAK) and incubated with primary antibodies for GADPH (1:1,000), FAK (1:1,000), p-FAK (1:500), and integrin β8 (1:500). The membranes were then incubated with horseradish peroxidase-conjugated anti-rabbit IgG (1:2,000). Proteins were visualized with Immobilon™ Western chemiluminescent HRP Substrate and imaged by DNR Bio-Imaging Systems (MF-ChemiBIS 3.2). Protein quantification was performed by measuring the average intensities of individual lanes using ImageJ software.

Immunofluorescent Staining. pMØ cells were coincubated with 10 μg mL^{-1} nGO-PEG for 24 h, and then fixed with 3.7% formaldehyde and treated with 0.1% triton for 5 min. After blocking with 1% BSA for 1 h, cells were then incubated with primary antibodies against β_8 integrin (1:200) and vinculin (1:200) overnight at 4 ^{o}C. Cells were next rinsed thrice and incubated with anti-rabbit IgG conjugated with Alexa Fluor®488 (1:500) and anti-mouse IgG conjugated with Alexa Fluor®647 (1:500) for 1 h. Finally, cells were rinsed with PBS and covered with a few drops of SlowFade®Gold Antifade Reagent to prevent fluorescence quenching.

For NF-κB immunofluorescence, the primary antibody p65 (1:200) and secondary antibody anti-rabbit Alexa Fluor®488 (1:500) were applied. 3 μg mL^{-1} DAPI was added 20 min before 1 h of incubation. After rinsing with PBS 3 times, the samples were examined under a TCS SP5 CLSM (Leica).

Quantigene detection. pMØ cells were seeded in a 96-well plate to reach 80% confluence. Here three groups were chosen: control, cells treated with nGO-PEG, and

cells with β_8-gene-silencing reagents and nGO-PEG. Quantigene detection was conducted after 12 h, 24 h, and 48 h of coincubation. Procedures were manipulated according to the instructions provided by manufacturers, and the final results were analyzed on a Luminex®200 (Luminex).

Experimental statistical analysis. Statistical evaluations of data were performed by Student's t test for two groups, and one way ANOVA for multiple groups. All results were expressed as mean ± standard deviation (SD). $P < 0.05$ was considered statistically significant.

Supplementary Movies S1 & S2: Trajectories of cells in the absence (S1) or presence (S2) of nGO-PEG (see main text for more description).

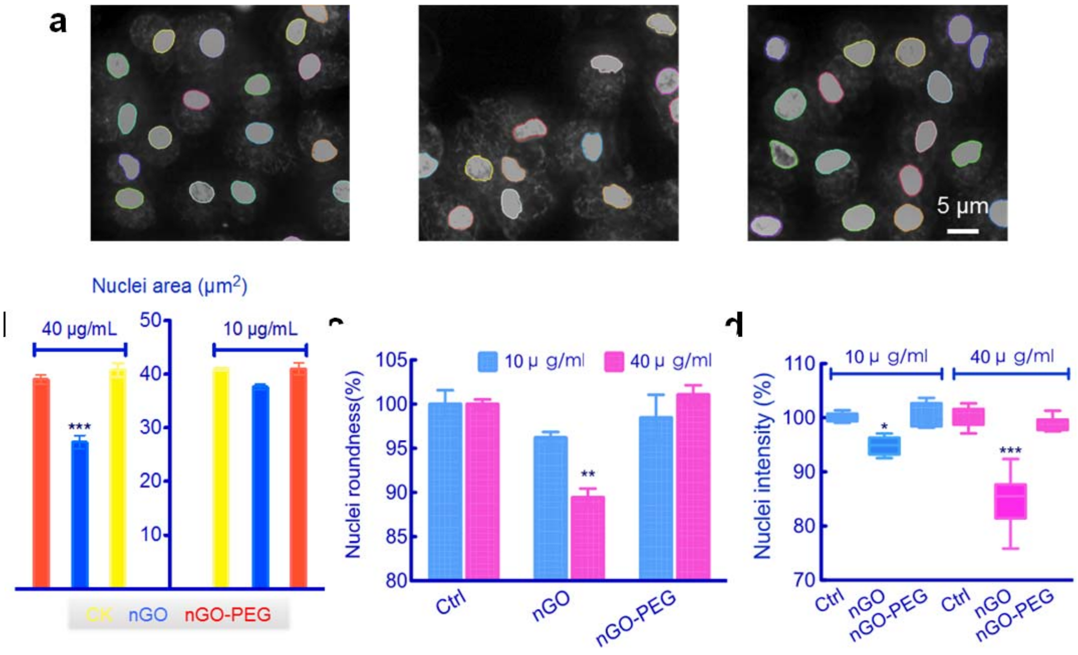

Supplementary Figure 1. Nuclear parameters measured using a Columbus analysis system after different nanomaterial treatments. (a) Effects of nGO and nGO-PEG on cell viability, as indicated by nuclear changes after 24 h of incubation. Nuclei were dyed with Hoechst and delineated with colorful contours using a high content screening system. (b) Nuclear area, (c) nuclear roundness, and (d) nuclear intensity. Macrophages treated with nGO-PEG exhibited nuclei characteristics very similar to those of normal cells. However, exposure to pristine nGO brought about varying degrees of nuclear changes, which generated noticeable damage at both low and high concentrations (10 and 40 µg mL^{-1}). Data are presented as means ± SD with $n = 3$. *$p < 0.05$, **$p < 0.01$ and ***$p < 0.001$.

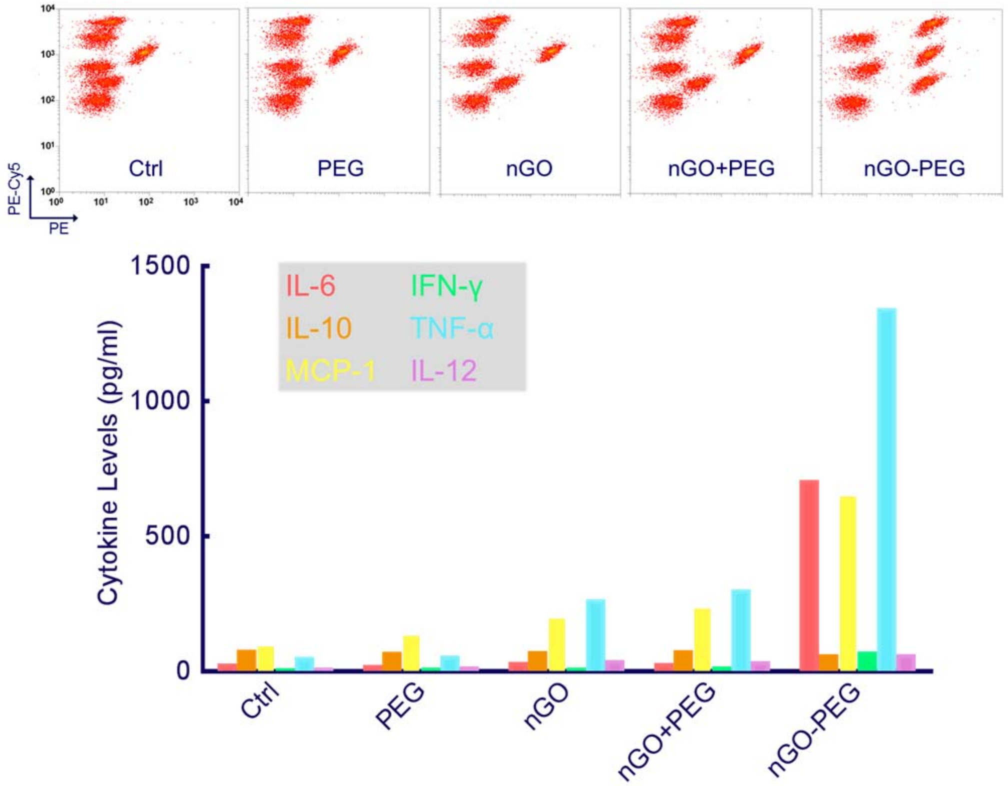

Supplementary Figure 2. Cytokine secretion induced by free PEG. Data are presented from free PEG, free nGO, a simple mixture of nGO and PEG, and nGO-PEG groups. Neither free PEG/nGO nor the nGO+PEG mixture could activate macrophages in a manner comparable to nGO-PEG. Therefore, both 2D structure and the chemical conjugation of PEG seem essential for the achieving high levels of cytokine secretion.

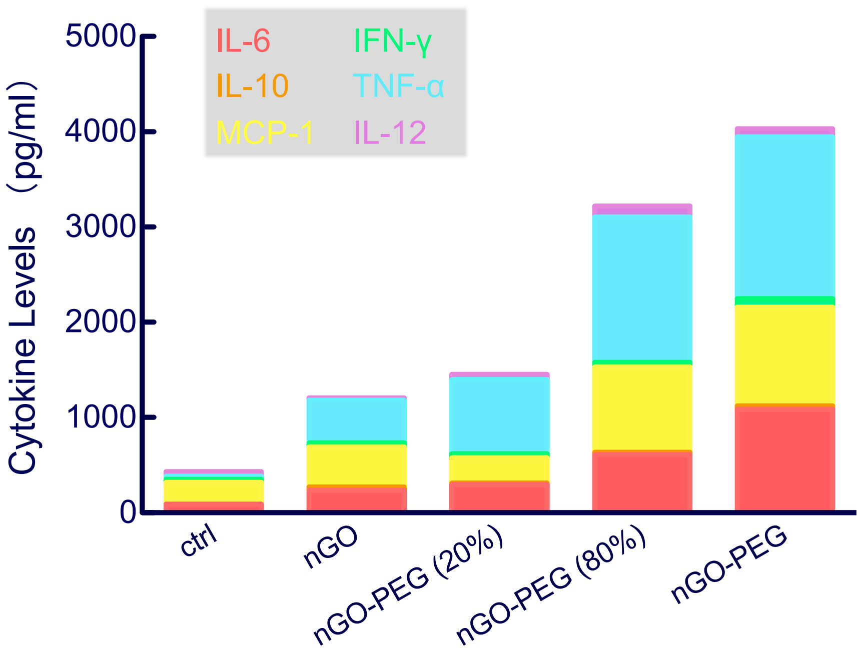

Supplementary Figure 3. Impact of PEGylation density on cytokine induction.

Cytokine secretion increases gradually with increasing PEG densities.

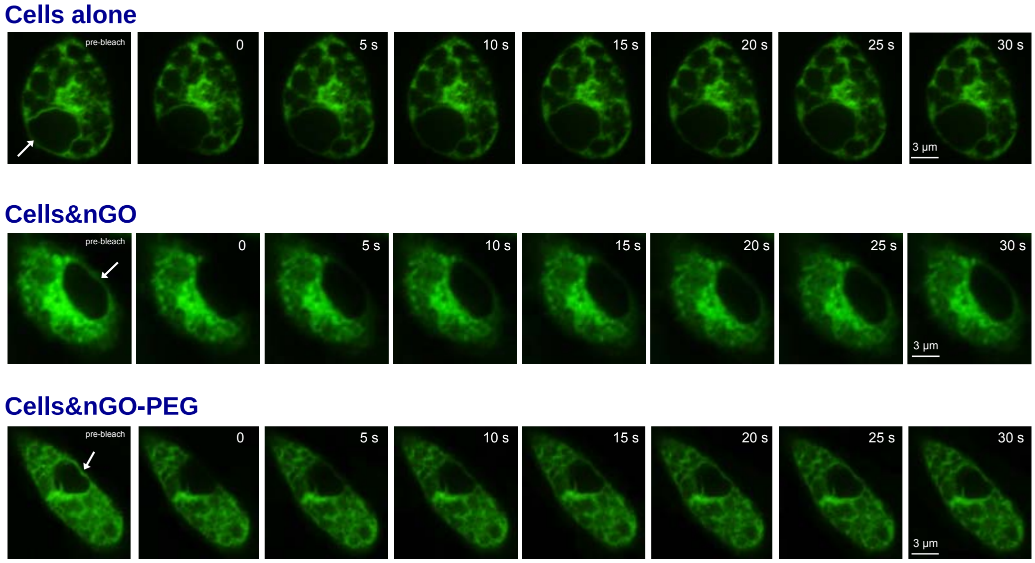

Supplementary Figure 4. Time-lapsed FRAP images of macrophages in the absence/presence of nGO and nGO-PEG. Cell membranes were stained with DiO, and the location of each photobleached spot is indicated with a white arrow. Recovery of the membrane in nGO-PEG stimulated cells was rapid, reaching near-normal fluorescence levels after ~20 s. Within the control and nGO-coincubation groups, fluorescence failed to recover after 30 s. Scale bar: 3 μm.

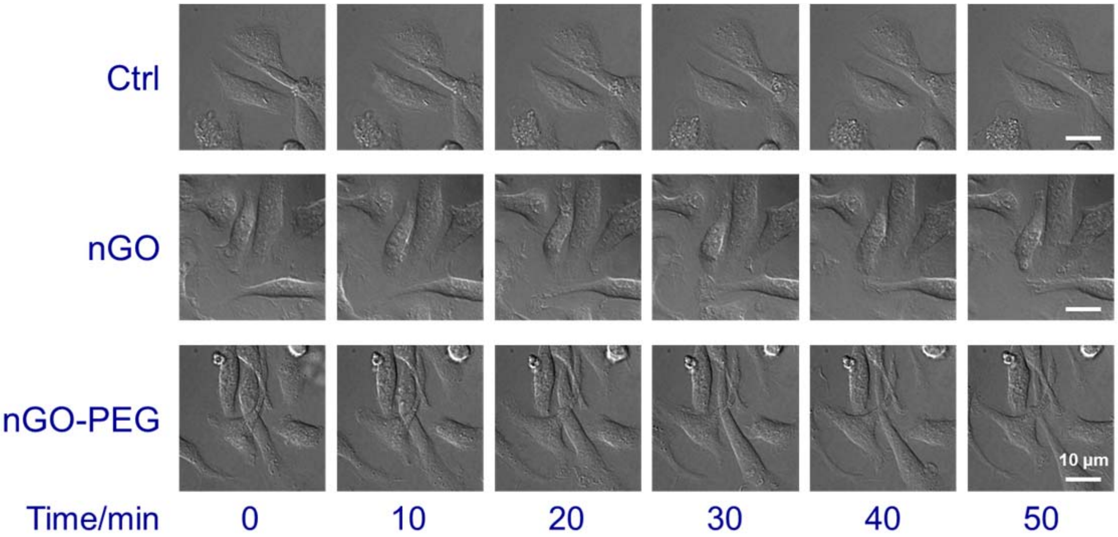

Supplementary Figure 5. Time lapsed monitoring of cell trajectories after treatment with nGO or nGO-PEG over 50 min. Over the observation time window, normal cells persisted in quiescent and inactive states, remaining at their original locations. NGO promoted cell migration to a small extent, while exposure to nGO-PEG greatly extended cells spheres of migration. Some of these mobile cells even moved outside of our field of vision over the monitoring period. Scale bar: 10 μm.

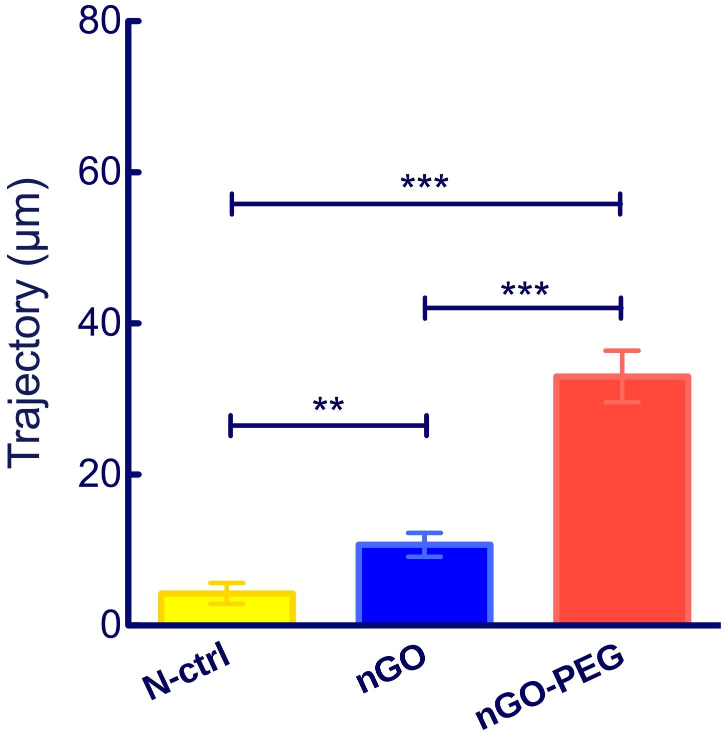

Supplementary Figure 6. Cell trajectory lengths in the absence/presence of nGO and nGO-PEG. Data are presented as means ± SD with n=6. **$p < 0.01$, ***$p < 0.001$.

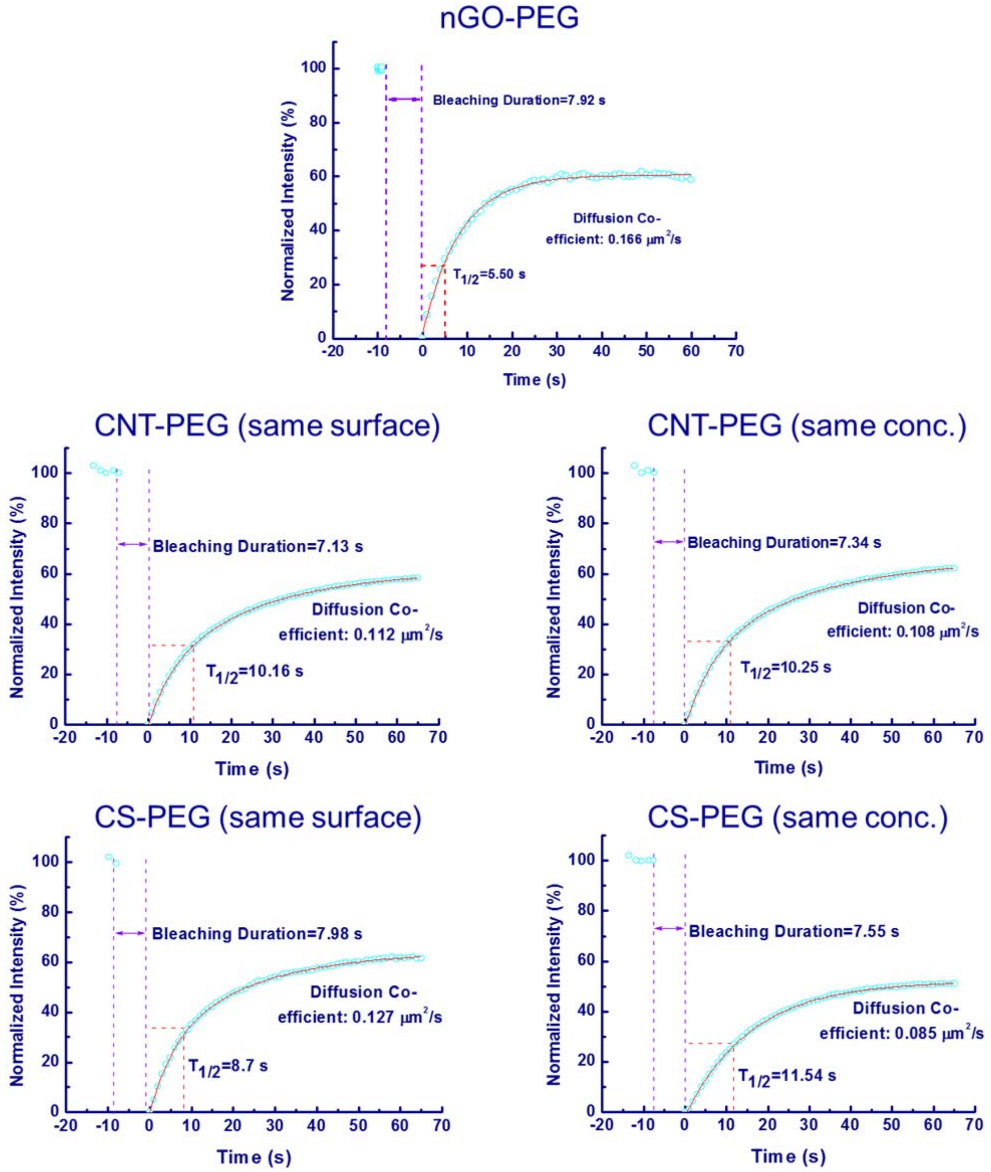

Supplementary Figure 7. Comparison of macrophage membrane FRAP kinetics. Data were collected after exposure to CS-PEG and CNT-PEG under similar constraints on surface area or dose (10 μg mL^{-1}). 2D nGO-PEG promoted most in membrane mobility among the three classes of nanomaterials studied. In this figure, "conc." is an abbreviation for concentration.

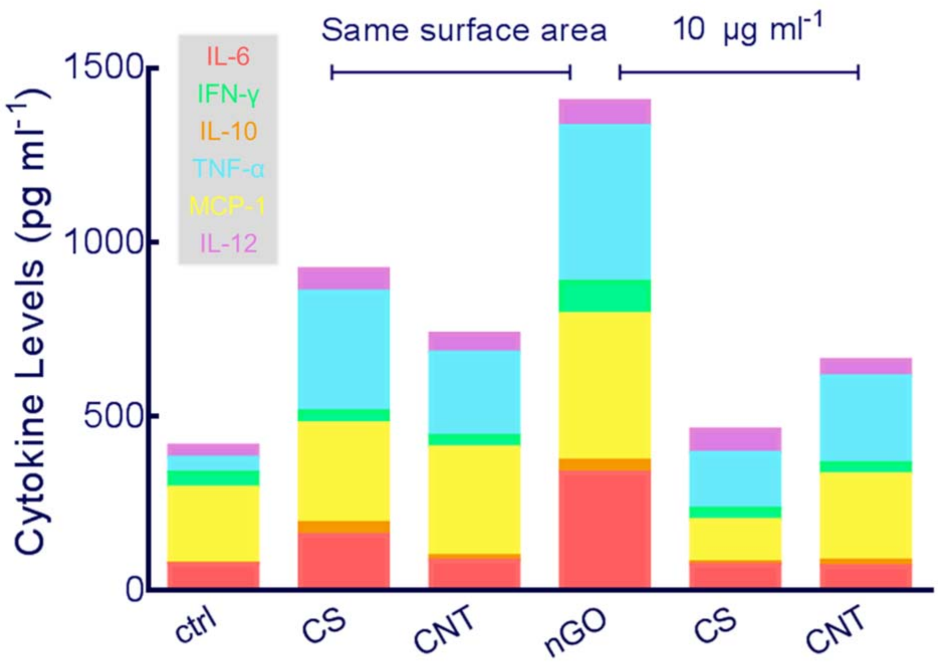

Supplementary Figure 8. **Macrophage cytokine secretion levels upon exposure to various pristine carbon nanomaterials**. Data are normalized by both surface area and concentration. Though uniformly diminished in extent, cytokine secretion levels induced by these nanomaterials conform to the trend seen in Fig. 3b. Each segment of the bar plot represents the mean of three replicas.

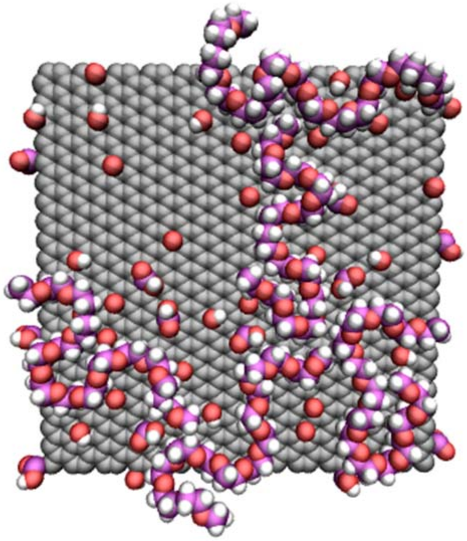

Supplementary Figure 9. Side view of PEGylated GO nanosheet. PEG carbons are represented in purple. Though PEG chains were initiated in fully extended configurations (radiating away from the nanosheet), the molecules quickly adsorbed onto the GO surface. Apart from transient desorption events near the free polymer termini, almost no unbinding events from the surface were observed over the course of simulation.

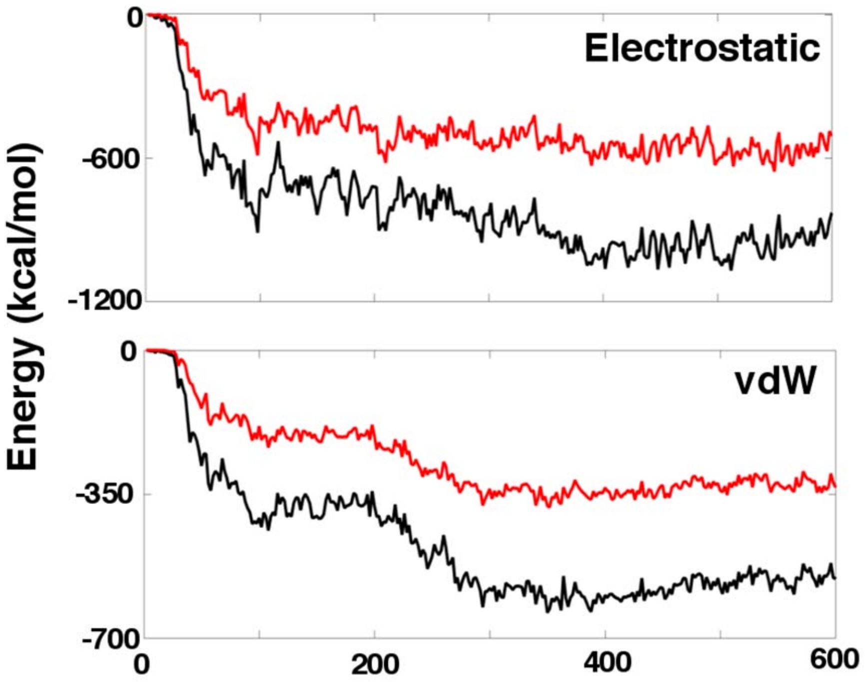

Supplementary Figure 10. Electrostatic and vdW interaction energies between membrane lipids and nGOs/nGO-PEGs placed in face-on configurations. The nGO-PEG system exhibits an interaction energy surplus owing to additional interactions between the membrane and GO-adsorbed PEG molecules; both electrostatic and vdW components of the interaction energy are enhanced by PEGylatation, which leads to interactions between the membrane and superficial PEG chains as well as buried PEG "anchors".

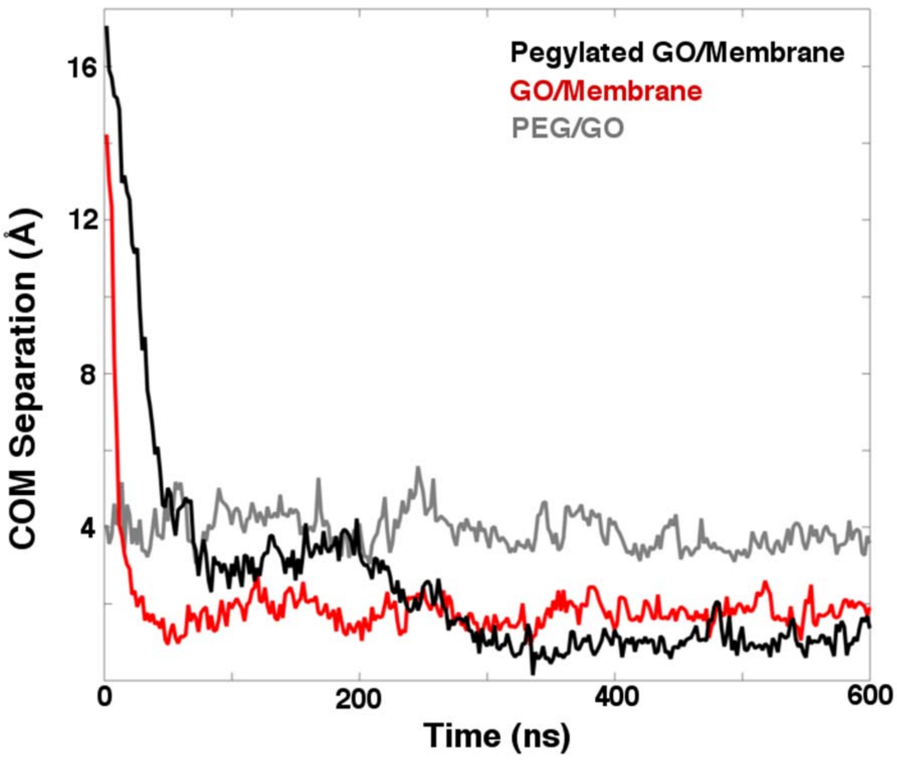

Supplementary Figure 11. Center-of-mass (COM) separation between PEGylated and bare nGOs in face-on configurations. Data are normalized to the extremity of the vdW radius of the lipid bilayer. As the figure indicates, both PEGylated and bare nGOs are quickly drawn onto the membrane surface. As the simulation proceeds, the PEGyated nGO is pulled even more tightly onto the membrane surface by the emergence of terminal PEG anchors. Overall, the COM separation between the GO nanosheet and its adsorbed PEG chains (grey curve) changes little over the course of the dynamics.

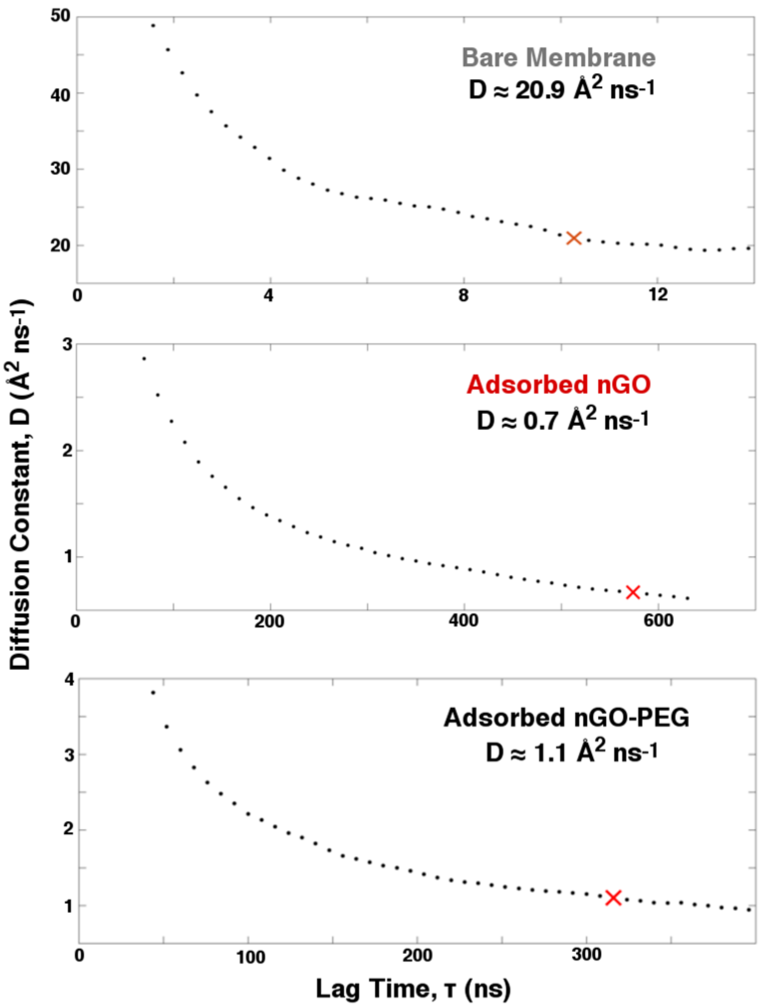

Supplementary Figure 12. Lipid head group diffusion constant calculations. Data correspond to lipids in an isolated membrane bilayer and in membranes to which face-on pristine and PEGylated nGOs have adsorbed. Though increased macrophage activation gives rise to observations of heightened membrane mobility in our experiments, diffusion in membrane regions directly contacting adsorbed nanosheets is expected to be arrested. Our simulations support this expectation: diffusion slows by a factor of approximately 20 after nanosheet adsorption. This phenomenon echoes reports of glass transitions seen in lipid membranes upon substrate binding. Diffusion constants are calculated as a function of the mean-square deviation over time; to confirm the Markov property in the diffusive dynamics, diffusion constant values are calculated as a function of a lag time window and reported at the threshold of (approximate) invariance with that parameter.

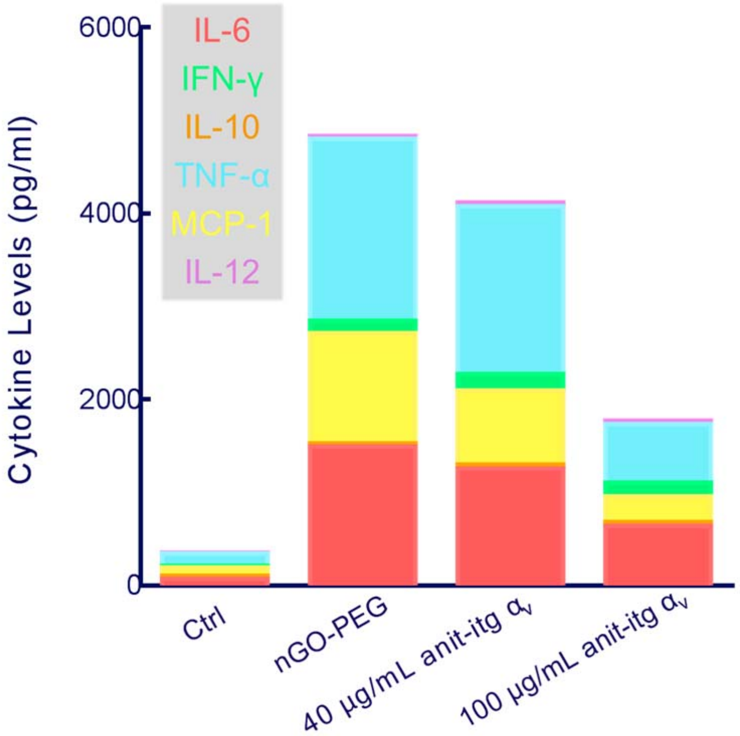

Supplementary Figure 13. Cytokine levels after integrin α_v blockage. Integrin α_v antibody at 40 μg mL^{-1} didn't completely inhibit the function of integrin α_v, but 100 μg mL^{-1} dramatically decreased cytokine secretion.

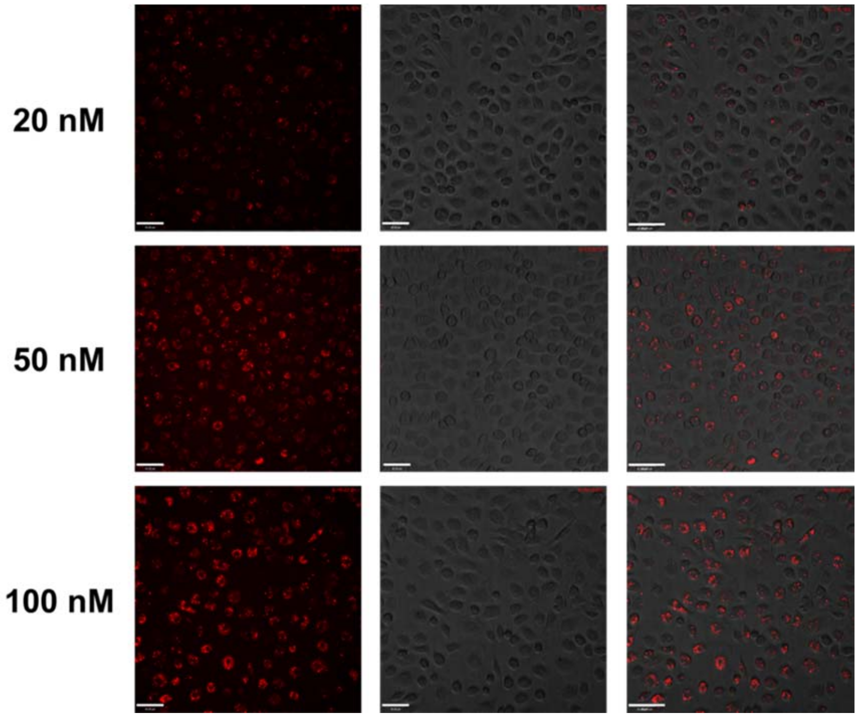

Supplementary Figure 14. Confocal images of siRNA-transfected cells using a red fluorescent positive control siRNA for 24 h. The overlaid images indicated that over 80% cells were transfected with siRNA at concentrations of 50 nM and 100 nM. Scale bar: 40 μm.

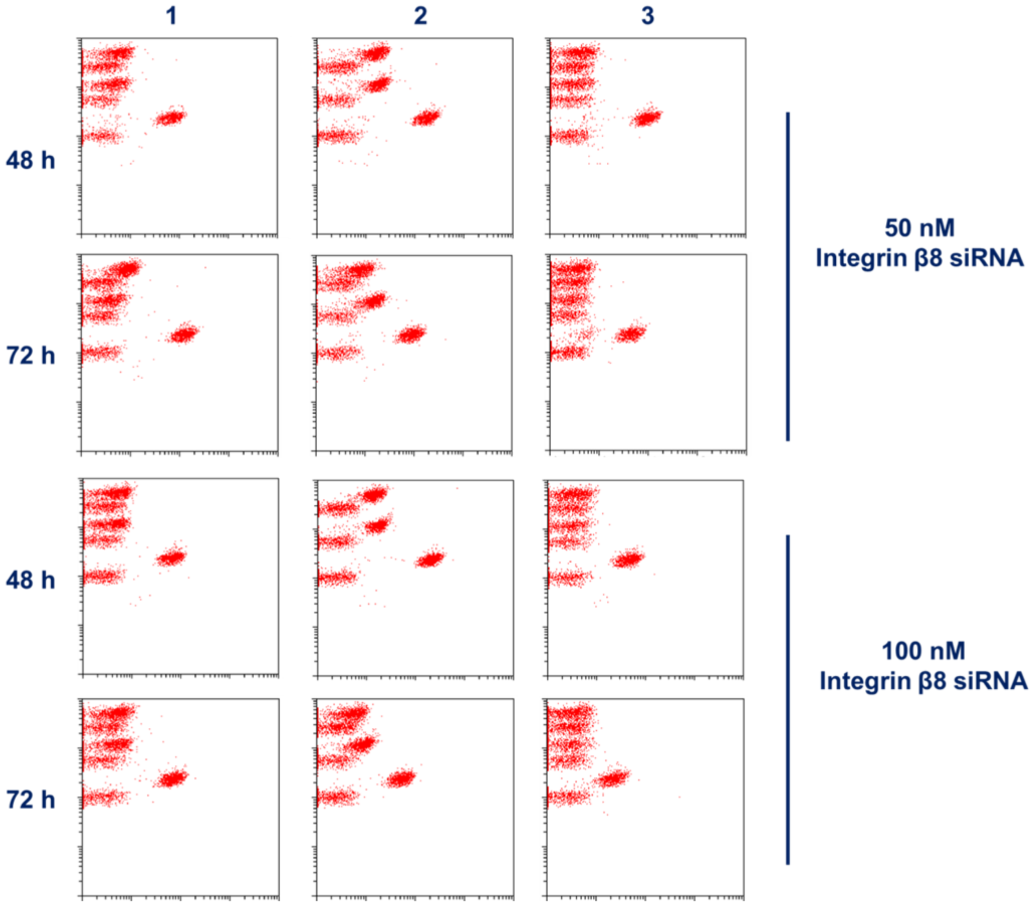

Supplementary Figure 15. Selection of the most effective integrin β_8 siRNA. No. 1, 2, 3 represent three different categories of Integrin β_8 siRNA. Their blocking efficiencies were evaluated by the degree of cytokine secretion observed over different coincubation conditions. The data show that the No.3 siRNA (No.MSS220603) performed best in all categories. Accordingly, a 48 h blocking period and 100nM dose were used in the following experiment.

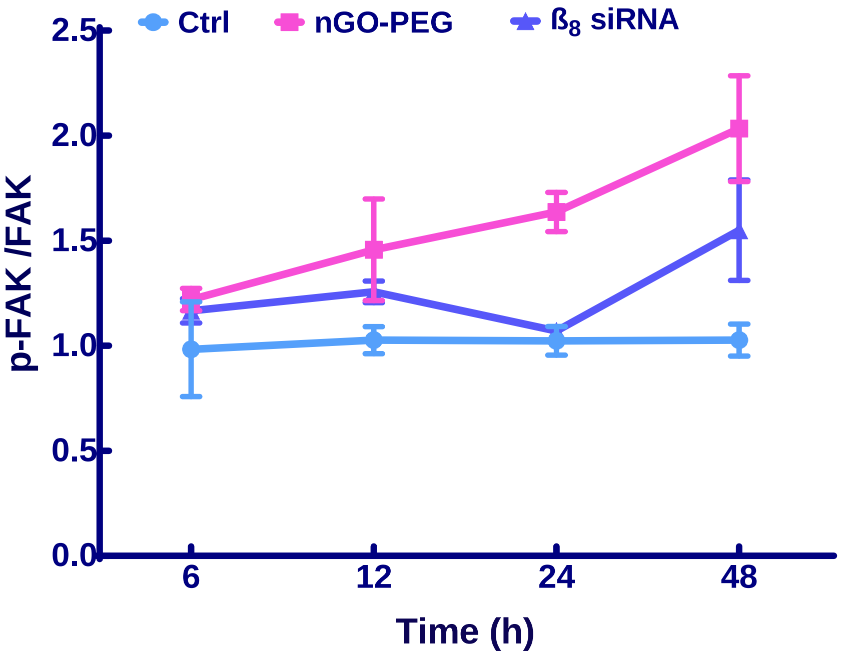

Supplementary Figure 16. Relative value of pFAK/FAK expression as a function of time. Measured by Western blot, FAK activation (p-FAK) started after 6 h and was distinct within 48 h. Treatment with integrin β_8 siRNA reduced the expression of p-FAK during the same period.

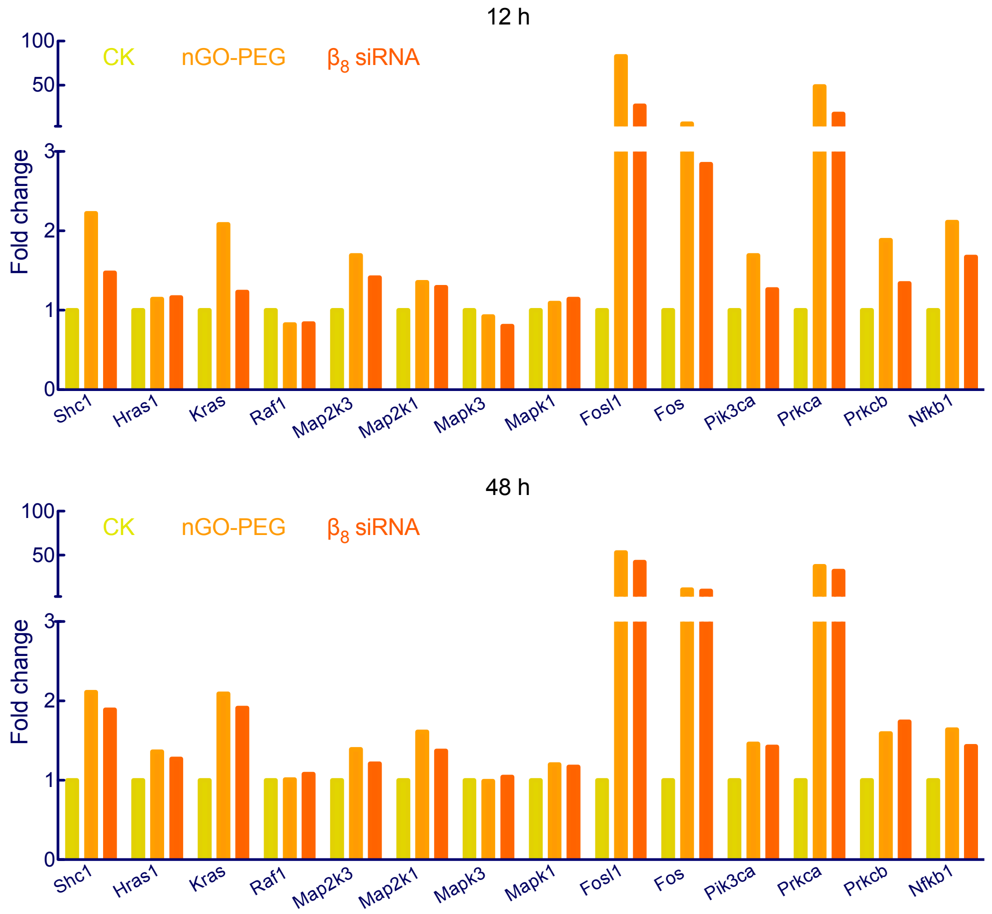

Supplementary Figure 17. Extent of gene expression changes in control/nGO-PEG/siRNA interference groups at 12/48 h. Changes were measured by by quantigene detection. The addition of nGO-PEG promoted the expression of related genes in comparison to untreated cells. Pretreatment with integrin β_8 siRNA slowed these changes.

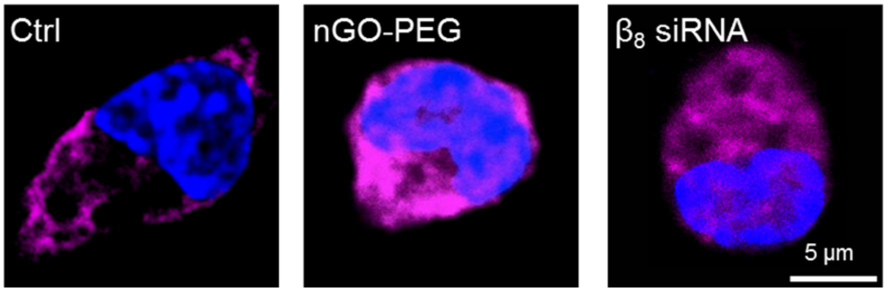

Supplementary Figure 18. Distribution of NF-κB observed in confocal images. nGO-PEG activated NF-κB, stimulating the transfer of its p65 subunit (purple) into the nucleus (blue). Silencing integrin β_8 gene with siRNA retarded this translocation.

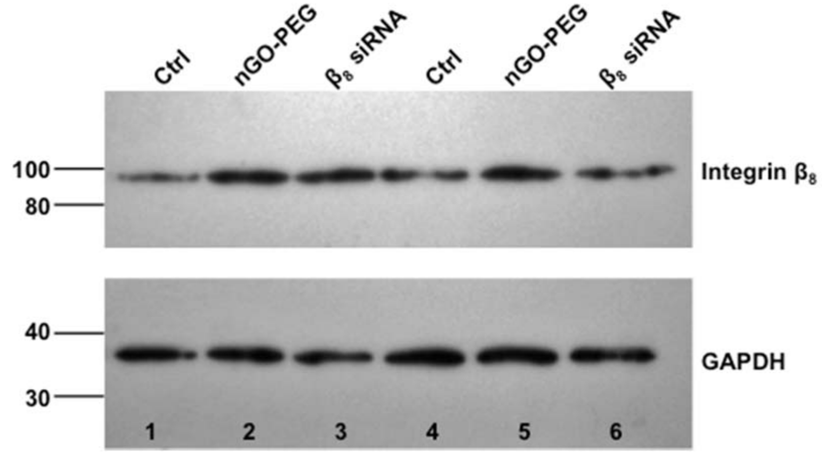

Supplementary Figure 19. Full gel and ladders for integrin β_8 and GAPDH. The Ctrl, nGO-PEG, and β_8 siRNA groups were duplicated. Lanes 4 and 5 are shown in Fig. 6c. Molecular weight markers are included in the left margin.

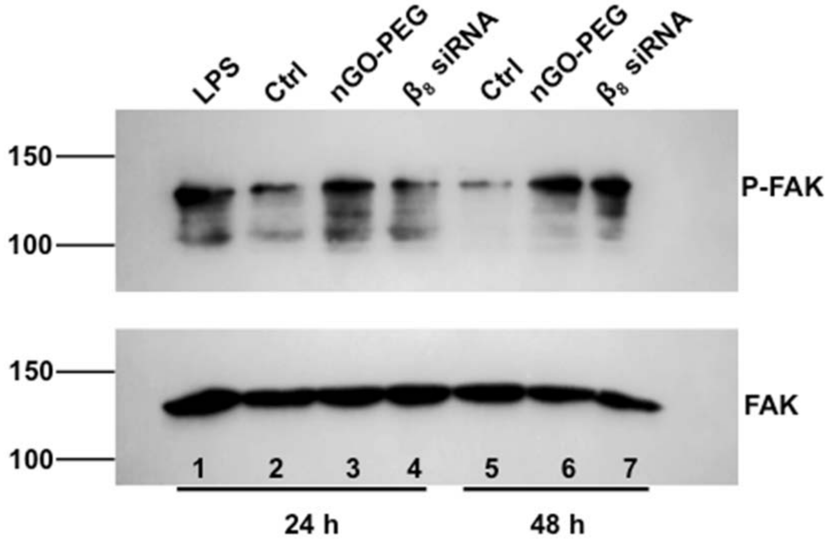

Supplementary Figure 20. Full gel and ladders for p-FAK and FAK under various treatment schemes at 24 h and 48 h progress. Lanes 2, 3, and 4 are shown in Fig. 6d. Molecular weight markers are included in the left margin.

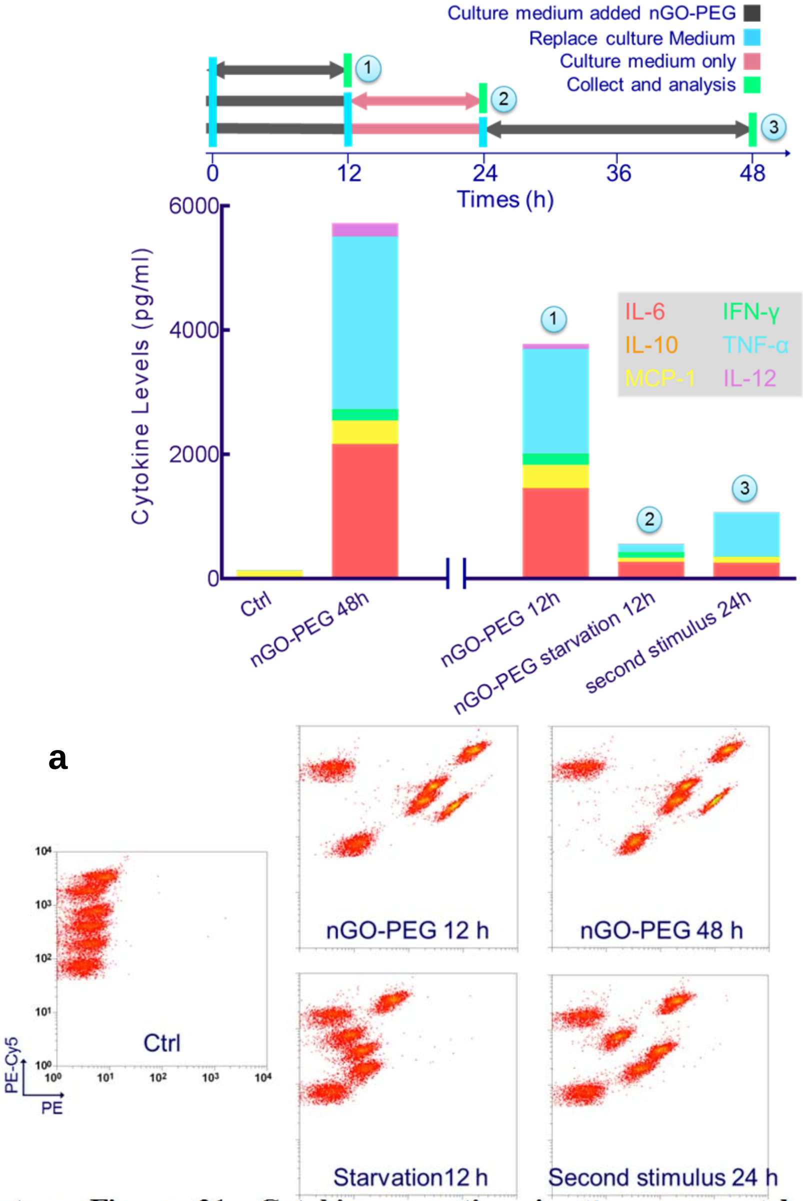

Supplementary Figure 21. Cytokine secretion in the presence/absence of nGO-PEG. (a) PMØ cells were stimulated with 10 μg mL^{-1} nGO-PEG solutions at the setting time (12 h, 24 h, 48 h). Cells were cultured with nGO-PEG for 12 h (point 1), at which point the nGO-PEG solution was replaced by fresh medium and recollected at 24 h (point 2). To investigate the response of cells upon second stimulus, fresh culture medium was removed again adding nGO-PEG-containing medium at 24 h, and then the results were analyzed at 48 h (point 3). After 12 h of nGO-PEG

starvation (point 2), cytokine secretion was suppressed. Cells receiving a second nGO-PEG stimulus after 12 h of deprivation (point 3) exhibited attenuated cytokine responses. (b) Corresponding FACS dotplots of cytokine secretion with/ without nGO-PEG.

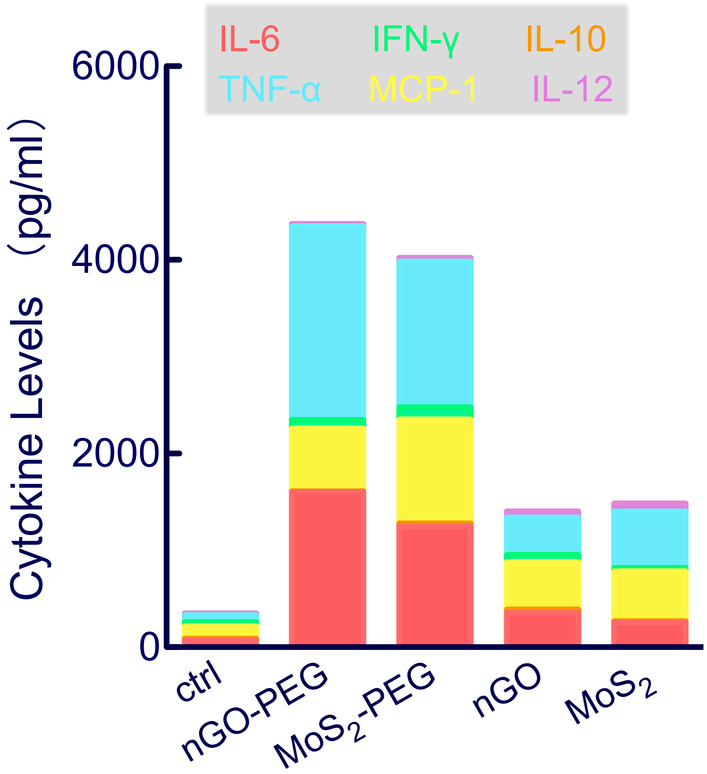

Supplementary Figure 22. Comparison of cytokine secretion levels between pristine/PEGylated nGO and MoS2 at 10 μg mL^{-1} nanomaterial concentrations. MoS2 and nGO stimulated comparable levels of cytokine secretion with and without PEGylation.

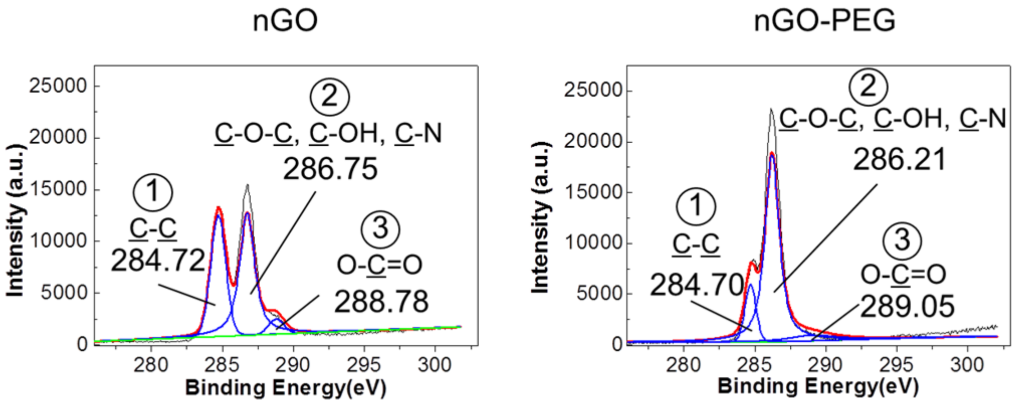

Supplementary Figure 23. Typical XPS C1s spectra for nGO and nGO-PEG. The binding energies of the peaks centered at around 284 eV (①), 286 eV (②) and 289 eV (③) are assigned to carbon atoms in C-C, C-O-C/C-OH/C-N and O-C=O, respectively. After deconvolution, the peak 2/peak 1 area ratio increased from 1.335 to 5.452, an effect mainly originating from the abundant C-O bonds in nGO-PEG. Considered alongside the near-neutral zeta potential in nGO-PEG, these results indicate that PEG chains were successfully conjugated to the GO surface.

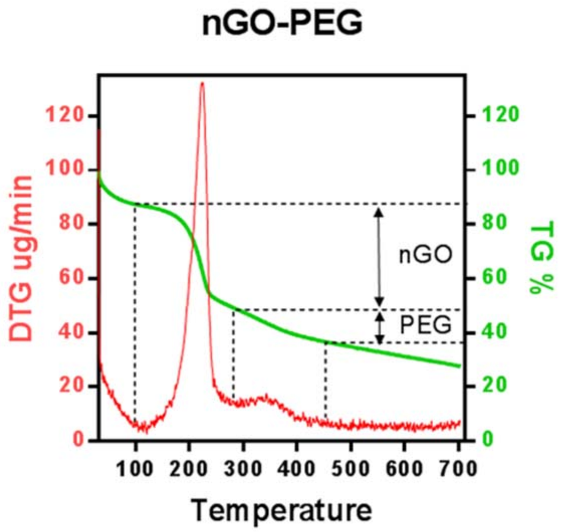

Supplementary Figure 24. Thermal gravimetry (TG) and differential thermal gravimetry (DTG) curves for nGO-PEG. The red DTG curve indicates ranges for the decomposition temperatures of nGO and PEG. Combining these DTG results with information from the TG curve, one sees that nGO undergoes an obvious weight loss (87.9% to 49.7%) above 100°C; the thermal decomposition of PEG occurs in a second step beyond ~300°C (49.7% to 36.6% weight change). Through the above-calculated proportions of nGO (38.2%) and PEG (13.1%), the mass ratio of nGO to PEG was determined to be about 3:1.

Term	Count	%	Pvalue
mmu04060:Cytokine-cytokine receptor interaction	77	3.61	4.23E-15
mmu04010:MAPK signaling pathway	56	2.63	8.22E-05
mmu04062:Chemokine signaling pathway	51	2.39	3.07E-08
mmu04510:Focal adhesion	34	1.59	0.0521

24 h KEGG Pathway			
Term	Count	%	Pvalue
mmu04060:Cytokine-cytokine receptor interaction	12	5.06	6.31E-04
mmu04514:Cell adhesion molecules (CAMs)	9	3.80	0.00149
mmu04510:Focal adhesion	8	3.38	0.0224
mmu04062:Chemokine signaling pathway	7	2.95	0.0450

48 h KEGG Pathway			
Term	Count	%	Pvalue
mmu04060:Cytokine-cytokine receptor interaction	24	5.31	1.06E-06
mmu04062:Chemokine signaling pathway	14	3.10	0.00352
mmu04010:MAPK signaling pathway	14	3.10	0.0602

Supplementary Table 1. Putative activated pathways, according to KEGG database, as a function of incubation time (12/24/48 h).

wt%	Mn	Cu	Fe
Before washing	1.1%	0.02%	0.4%
After washing	0.01%	ND	ND

Supplementary Table 2. Heavy metal content of GO samples before and after washing.

	nGO	nGO-PEG	CS	CS-PEG	CNT	CNT-PEG
ζ Potential (mV)	-34.01 ± 1.50	-7.89± 1.67	-43.80 ± 0.50	-8.71 ± 2.16	-55.0 ± 1.20	-6.95 ± 1.05

Supplementary Table 3. Zeta potential of carbon materials before and after PEGylation. PEG reduced each potential to near neutrality. Data are means ± SD, with n = 3.

Supplementary References

1. Humphrey, W., Dalke, A. & Schulten, K. VMD: Visual molecular dynamics. *J. Mol. Graph. Model.* **14**, 33-38 (1996).
2. He, H.Y., Klinowski, J., Forster, M. & Lerf, A. A new structural model for graphite oxide. *Chem. Phys. Lett.* **287**, 53-56 (1998).
3. Vorobyov, I. et al. Additive and classical drude polarizable force fields for linear and cyclic ethers. *J. Chem. Theory Comput.* **3**, 1120-1133 (2007).
4. Tu, Y. et al. Destructive extraction of phospholipids from Escherichia coli membranes by graphene nanosheets. *Nat. Nanotechnol.* **8**, 594-601 (2013).
5. MacKerell, A.D., Banavali, N. & Foloppe, N. Development and current status of the CHARMM force field for nucleic acids. *Biopolymers* **56**, 257-265 (2001).
6. Phillips, J.C. et al. Scalable molecular dynamics with NAMD. *J. Comput. Chem.* **26**, 1781-1802 (2005).
7. Major, J., Fletcher, J.E. & Hamilton, T.A. IL-4 pretreatment selectively enhances cytokine and chemokine production in lipopolysaccharide-stimulated mouse peritoneal macrophages. *J. Immunol.* **168**, 2456-2463 (2002).
8. Yue, H., Wei, W., Yue, Z., Wang, B., Luo, N. et al. The role of the lateral dimension of graphene oxide in the regulation of cellular responses. *Biomaterials*, **33**, 4013-4021 (2012).
9. Liu, Z., Robinson, J.T., Sun, X. & Dai, H. PEGylated nanographene oxide for delivery of water-insoluble cancer drugs. *J. Am. Chem. Soc.* **130**, 10876-10877 (2008).
10. Sun, X. & Li, Y. Colloidal carbon spheres and their core/shell structures with noble-metal nanoparticles. *Angew. Chem., Int. Ed.* **43**, 597-601 (2004).
